# Physical Activity’s Role in Reducing Neuropsychiatric Symptoms Among Older Adults with Dementia

**DOI:** 10.1093/geroni/igaf122.2171

**Published:** 2025-12-31

**Authors:** Lin-Na Chou, Niying Li, Yongseop Kim, Anne Thackeray

**Affiliations:** University of Utah, Saratoga Springs, Utah, United States; University of Georgia, Athens, Georgia, United States; University of Utah, University of Utah, Utah, United States; University of Utah, Salt Lake City, Utah, United States

## Abstract

Neuropsychiatric symptoms (NPS) pose significant challenges in dementia care. With limited pharmacological treatments, identifying behavioral targets is crucial for developing effective interventions. This study examines the associations between Alzheimer’s Disease and Related Dementia (ADRD) and physical activity (PA) with NPS among individuals aged 70 years and older, using data from the 2021 National Health and Aging Trends Study (NHATS). NPS assessed included depression (PHQ-2), anxiety (GAD-2) and sleeping problems (single-item question on difficulty falling asleep). ADRD was determined using the NHATS algorithm, and PA was measured through self-reported questions on walking for exercise and vigorous activities in the last month. Data from 3388 subjects (21% with ADRD) were analyzed. In multivariable logistic regression models adjusted for sociodemographic factors and medical conditions, ADRD was associated with increased risks of depression (OR, 95% CI: 2.05, 1.41-2.97) and anxiety (2.69, 1.76-4.11). Walking was associated with decreased risks of depression (0.70, 0.51-0.97) and anxiety (0.67, 0.46-0.97), while vigorous activities were associated with decreased risks of depression (0.59, 0.40-0.87) and sleep problems (0.70, 0.52-0.94). Subgroup analysis among the oldest old adults (age ≥85 years) revealed that walking reduced the risk of anxiety (0.52, 0.27-0.99). This study underscores the benefits of walking and vigorous activities in reducing NPS risk. Incorporating PA into regular care practices is highly encouraged for healthcare providers and caregivers to enhance the well-being of individuals with dementia.

